# The biochemical differentiation of *Enterobacter sakazakii *genotypes

**DOI:** 10.1186/1471-2180-6-94

**Published:** 2006-10-26

**Authors:** Carol Iversen, Mike Waddington, Jim J Farmer, Stephen J Forsythe

**Affiliations:** 1 School of Biomedical and Natural Sciences, Nottingham Trent University, Clifton Lane, Nottingham, NG11 8NS, UK; 2 Accugenix, 223 Lake Drive, Newark, DE 19702, USA; 3 Silver Hill Associates, 1781 Silver Hill Rd, Stone Mountain, GA 30087, USA; 4 Quality and Safety Assurance Department, Nestlé Research Center, PO Box 44, CH 1000 Lausanne 26, Switzerland

## Abstract

**Background:**

*Enterobacter sakazakii *is an emergent pathogen that has been associated with neonatal infections through contaminated powdered infant milk formula. The species was defined by Farmer *et al. *(1980) who described 15 biogroups according to the biochemical characterization of 57 strains. This present study compares genotypes (DNA cluster groups based on partial 16S rDNA sequence analysis) with the biochemical traits for 189 *E. sakazakii *strains.

**Results:**

Analysis of partial 16S rDNA sequences gave 4 well defined phylogenetic groups. Cluster group 1 was composed of the majority of strains (170/189) and included Biogroups 1–5, 7–9, 11, 13 and 14. Cluster 3 corresponded with Biogroup 15 and cluster 4 with Biogroups 6, 10 and 12. Cluster group 2 comprised a new Biogroup 16. For the isolates in this study, the four DNA cluster groups can be distinguished using the inositol, dulcitol and indole tests.

**Conclusion:**

This study demonstrates an agreement between genotyping (partial 16S rDNA) and biotyping and describes a new biogroup of *E. sakazakii*.

## Background

*Enterobacter sakazakii *is an emergent pathogen that is associated with neonatal infection [1,2]. Most reported outbreaks have occurred in neonatal intensive care units and some have been linked to the ingestion of contaminated powdered infant milk formula [3,4].

*E. sakazakii *was defined as a new species by Farmer *et al*. 1980. Using DNA-DNA hybridization the previously 'yellow pigmented *Enterobacter cloacae*' was shown to be 41–54% related to *Enterobacter *and *Citrobacter *species. Farmer *et al. *[5] described 15 biogroups of *E. sakazakii *based on biochemical profiles with the wild type Biogroup 1 being the most common. Since 1980 prokaryotic systematics has changed with the increasing use of 16S rDNA sequence analysis [6,7]. Harada and Ishikawa [8] used DNA sequence analysis of the *gro*EL operon to determine the phylogenetic relationship among *Enterobacter*, *Pantoea*, *Klebsiella*, *Serratia *and *Erwinia *species. Hoffmann and Roggenkamp [9] used *hsp*60 (*gro*EL homologue) DNA sequence variation to investigate *E. cloacae *polyphyletic groups. Previously, it has been shown by both partial 16S rDNA and *hsp*60 gene sequencing that isolates identified as *E. sakazakii *using commercial identification kits form at least four distinct clusters [10]. Recently, an Artificial Neural Network has been published which identified key biochemical and 16S rDNA sequences that distinguish *E. sakazakii *from closely related organisms [11].

In this study, we compare the biogroups of 189 strains with the four 16S rDNA cluster groups. We also identified key biochemical characteristics for the differentiation of these four genotypes. This significantly extends the previous work of Farmer et al. [5], which used five strains to genetically define the species and a total of 57 strains to define biogroups.

## Results

### Biogroups

The biochemical profiles obtained for each strain were compared to the biogroups originally described by Farmer *et al *[5]. Clinical strains were distributed among fourteen of the biogroups (Table 1). The defining tests were motility, Voges-Proskauer, methyl red, indole, ornithine decarboxylase, reduction of nitrate to nitrite, production of gas from D-glucose, malonate utilization and production of acid from myo-inositol and dulcitol. Where strains could not be assigned to an original biogroup, a new biogroup or subgroup was designated (Table 2). The majority of isolates (60/189) were in Biogroup 1 with the *E. sakazakii *ATCC 29544^T ^type strain. These strains were motile, produced gas from glucose, produced acid from inositol, reduced nitrate and were positive for Voges-Proskauer and ornithine decarboxylase, but negative for methyl red, indole, malonate utilization and acid production from dulcitol. Biogroup 2 (n = 42) contained isolates negative for acid production from inositol; four of these were also non-motile. Biogroup 3 (non-motile) contained six strains and Biogroup 4 (ornithine negative) contained nine strains three of which were non-motile. Biogroup 5 (n = 16) was positive for malonate utilization and six of these were non-motile. Biogroup 6 (n = 2) was positive for indole and Biogroup 7 (n = 4) was negative for gas production from glucose. Biogroup 8 (n = 7) was defined by the inability to reduce nitrate. Two of these 7 strains were positive in the malonate test, three were negative for the inositol test and two were inositol negative but malonate positive. Biogroup 9 contained 13 strains that were inositol negative and malonate positive. Biogroup 10 contained one strain that was inositol negative and indole positive, while Biogroup 11 contained one strain that was inositol negative and dulcitol positive. Biogroup 12 was also represented by only one strain, which was indole and malonate positive. The seven isolates in Biogroup 13 were negative for the Voges-Proskauer reaction, three were non-motile, one was negative for methyl red and one was negative for ornithine. Biogroup 14 (n = 5) was negative for ornithine decarboxylase and inositol, with four of these strains being positive for malonate. Biogroup 15 was positive for all the tests performed except methyl red. A new group (Biogroup 16) had to be defined to accommodate 9 strains which were inositol and dulcitol positive, but indole negative. They were malonate positive, with the exception of one strain. Two strains were non-motile and one of these was also ornithine decarboxylase negative. Acid production from α-methyl-D-glucoside was included in the original study [1] and all biogroups were reported positive for this trait with the exception of Biogroup 15. As Biogroup 15 could be distinguished from the other biogroups without the α-methyl-D-glucoside test, this was not repeated for all strains in this study.

### Phylogenetic analysis

**Figure 1:**
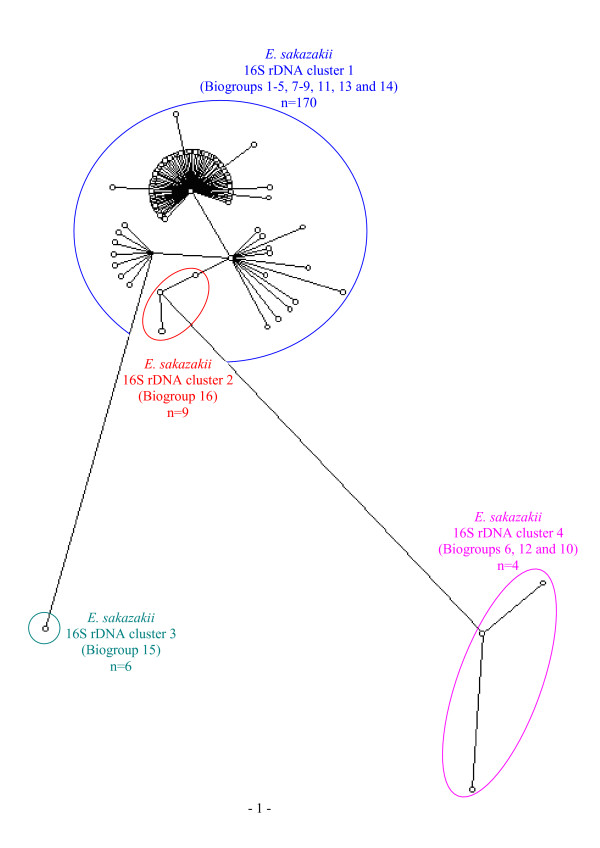
**Maximum parsimony tree (unrooted) showing the four genomic clusters of *E. sakazakii *based on partial 16S rDNA sequence analysis**. Gaps were not considered an extra state, and the topology was optimised using simulated annealing.

Partial 16S rDNA sequences (528 bp) containing less than 1% undetermined positions were obtained for all strains in this study. The sequences were analysed using the maximum parsimony method, which is an evolutionary model that searches for the simplest tree that can be constructed using the fewest inferred changes between characters. The topology was optimised using simulated annealing, a heuristic that occasionally accepts a worse tree during the course of the search allowing it to escape local optima. This method is more economical than the more usual heuristic searches (stepwise addition and hill-climbing), which can require many randon re-starts, especially with large data matrices [12]. In agreement with previous reports [10], the isolates used in this study were divided into four genomic groups by 16S rDNA sequence analysis (Fig 1). The majority (170/189) of the presumptive *E. sakazakii *isolates in this study clustered with the type strain (Fig. 1*E. sakazakii *cluster 1). Cluster 1 was composed of the majority of biogroups, Biogroups 1–5, 7–9, 11, 13 and 14. The 9 isolates forming the new proposed Biogroup 16 corresponded with *E. sakazakii *cluster 2 (Fig. 1 cluster 2), with between 1.23–1.89% 16S rDNA sequence difference from the *E. sakazakii *type strain. No strains representative of this biochemical profile were included in the original study by Farmer *et al*. [5].

The six isolates in Biogroup 15 corresponded to *E. sakazakii *cluster 3 (Fig. 1 cluster 3). The four strains described as *E. sakazakii *cluster 4 (Fig. 1 cluster 4) represent Biogroups 6, 10 and 12. For this dataset, the four partial 16S rDNA cluster groups can be distinguished biochemically using the indole, dulcitol, and inositol tests (Table 4). Cluster 1 strains are variable for inositol, negative for indole, and dulcitol; with the exception of Biogroup 11, which is dulcitol positive and inositol negative. Cluster 2 – Biogroup 16 strains are positive for inositol and dulcitol but negative for indole. Cluster 3 – Biogroup 15 is positive for inositol, dulcitol and indole. Cluster 4 strains were also positive for indole but can be distinguished from Cluster 3 as they are negative for dulcitol. One of the strains in Cluster 4 was inositol negative (Biogroup 10) and one was malonate positive (Biogroup 12). There were insufficient isolates in Cluster 4 biogroups to determine whether these biogroups could be further genomically divided.

## Discussion

Farmer *et al. *[5] described 15 biogroups of 57 strains of *E. sakazakii*. Five strains were used to genetically define the species by DNA-DNA hybridization and the remaining strain definitions were phenotypic. This study defines isolates in terms of both phenotype and genotype and extends the initial 1980 [5] study with the analysis of 189 strains. According to partial 16S rDNA sequence analysis the majority of *E. sakazakii *strains clustered with the type strain (Figure 1), and within this cluster there was no clear further genomic division corresponding to biogroup. A second cluster of closely related strains was identified (cluster 2) which were biochemically distinct from cluster 1 and formed a biogroup that had not been previously described by Farmer *et al *[5]. These strains were not assigned a species match using the MicroSeq 500 or supplemental bacterial databases, but the nearest match was *E. sakazakii *(1.23–1.89%). Biogroup 15 [5] corresponds to *E. sakazakii *cluster 3, while Biogroups 6, 10 and 12 correspond to *E. sakazakii *cluster 4 [10,11].

Analysis of the full 16S sequence of strains corresponding to cluster groups 2 (biogroup 16) [13] and 3 (biogroup 15) [14] show these are less than 3% divergent from cluster group 1. Although DNA-DNA hybridization is the acknowledged standard for species delineation this technique was beyond the scope of this study, which focuses on evaluation of the biogroups reported when *E. sakazakii *was originally described. As well as defining a new biogroup (with three subgroups), this study found 10 subgroups within the original biogroups. The malonate and motility tests account for the majority of the subdivisions of the original biogroups.

In this study, the majority of food isolates belonged to Biogroups 1 and 2. All biogroups except 10 and 16 contained at least one strain from a clinical source. The greatest number of clinical strains (10 isolates) was found to belong to Biogroup 9 (Table 1). However, this included 9 isolates out of the ten from the same hospital. Therefore this may not be an indication of increased pathogenicity of this biogroup, but is likely due to an over representation of one clonal type from a single source. Most of the food isolates (including infant foods) belonged to Biogroups 1 and 2.

## Conclusion

Biogroup 1 was the major group with approximately one third of the *E. sakazakii *strains (60/189). Cluster 1 was the major DNA cluster (170/189 strains), and was composed of Biogroups 1–5, 7–9, 11, 13 and 14. Cluster 3 is equivalent to Biogroup 15, and a new Biogroup 16 was designated for the strains in Cluster 2. For this dataset it is possible to differentiate the four genomic clusters using indole, dulcitol and inositol tests.

## Methods

### Sources of bacterial strains

A total of 189 *E. sakazakii *strains were analyzed in this study. Strains were from diverse food, clinical and environmental sources worldwide. The origin distribution of the isolates is given in Table 1. At least one strain from each of the biogroups described when the *E. sakazakii *species was designated were included and representative strains for each biogroup are given in Table 3. The type strain (NCTC 11467^T^) was purchased from the NCTC, London, UK, as were strains NCTC 9844, NCTC 9238 and NCTC 9846. Strain NCIMB 8272 was purchased from the NCIMB, Aberdeen, Scotland. Eleven strains, CDC 5960-70, 1895-73, 0743-75, 3523-75, 9363-75, 9369-75, 0407-77, 0996-77, 1058-77, 1716-77, and 3128-77 were donated by the CDC, Atlanta, GA, USA. Strains were also kindly donated by the following: – Nestlé Research Center, Lausanne, Switzerland; Health Products and Food branch, Health Canada; Children's Hospital Los Angeles CA, USA; Northern Foods, UK; Oxoid Ltd., Basingstoke, UK; Hospital Cèské Budéjovice, Czech Republic; Institut fûr Tierärztliche Nahrungsmittelkunde Milchwissenschaften, Justus-Liebig-Universität Gießen, Germany; Nottingham City Hospital Trust, Nottingham, UK and the Department of Medical Microbiology, Radboud, Nijmegen, Netherlands. All other strains were food and environmental isolates from the culture collection at Nottingham Trent University, Nottingham, UK.

### Phenotypic characterisation

In addition to phenotypes derived from commercial biochemical test kits (Microbact GN2, Oxoid; API 20E and ID32E, BioMérieux) according to manufacturers' recommendations, the following tests were performed using conventional manual methods. Motility was determined at 37°C after 24 and 48 h using motility medium (tryptose 10 g l^-1^, NaCl 5 g l^-1^, agar 5 g l^-1^, pH 7.2 ± 0.2). Acid production from carbohydrates was tested in phenol red broth base (10 g l^-1 ^peptone, 1 g l^-1 ^yeast extract, 5 g l^-1 ^NaCl, 0.018 g l^-1 ^phenol red) with the addition of filter-sterilized carbohydrate solution (final concentration 0.5%). Gas production from D-glucose was determined by collection in Durham tubes. Malonate utilization was determined using sodium malonate broth (sodium malonate 3 g l^-1^, ammonium sulphate 2 g l^-1^, bromothymol blue 0.025 g l^-1^, NaCl 2 g l^-1^, yeast extract 1 g l^-1^, dipotassium hydrogen phosphate 0.6 g l^-1^, potassium dihydrogen phosphate 0.4 g l^-1^). The methyl red test was performed by addition of indicator (0.1 g methyl red per 300 ml 95% ethanol) to cultures grown for 48 h in 4 ml of MR-VP broth (VWR, 1.05712.0500). The Voges-Proskauer test was performed by the addition of 40% potassium hydroxide in water and 5% 1-naphthol in (95% ethanol) to cultures grown for 24 hours in MR-VP broth. Indole production was measured by the addition of Kovacs reagent (5 g p-dimethylaminobenzaldehyde, 25 ml HCl, 75 ml pentanol-1-ol) or James Reagent (70542 BioMérieux) to cultures grown for 24 h in peptone water (CM0009 Oxoid). Nitrate reduction to nitrite was determined by the addition of 1% sulphanilamide in 1 M HCl and 0.02% N-1 naphthylene diamine HCl in water to nitrate test broths. Zinc dust was added to negative tubes of nitrate-negative strains to confirm the presence of unreduced nitrate.

### Genotypic characterisation

Comparative 16S rDNA sequencing (528 bp) was performed as previously described [11] by Accugenix (Newark, DE, USA) using the MicroSeq™ 500 16S rDNA Bacterial Sequencing Kit (Applied Biosystems).

A maximum parsimony tree (Fig. 1) of 16S rDNA sequences was generated using Bionumerics (Applied Maths, Belgium). Gaps were not considered an extra state and the topology was optimized using simulated annealing.

## Authors' contributions

CI carried out the biochemical tests, analyzed the data and wrote the initial manuscript. MW provided the 16S rDNA sequencing, assembly and editing.

JJF and SJF edited the manuscript; SJF managed the project. All authors read and approved the final manuscript.

## Figures and Tables

**Table 1:** Origins of *E. sakazakii *isolates used in this study.

Biogroup	Strain origin					Total
		
	Clinical	Powdered infant formula and infant food	Other dried food products	Environmental	Unknown	
1	7	16	33	2	2	60
2	4	12	23		3	42
3	4		1		1	6
4	4	1	1		3	9
5	2	2	11		1	16
6	1		1			2
7	2		1		1	4
8	1	2	2		2	7
9	10	2	1			13
10					1	1
11	1					1
12	1					1
13	3		3		1	7
14	1	2	2			5
15	1		3		2	6
16		1	7	1		9

Total	42	38	89	3	17	189

**Table 2:** Assignment of strains to the biogroups originally defined by Farmer *et al. *[1]

Farmer Biogroup	Additional group	Phenotype^a^										No. of strains	16s rDNA cluster
				
		VP	MR	Nit	Orn	Mot	Ino	Dul	Ind	Malo	Gas		
1		+	-	+	+	+	+	-	-	-	+	60	1
2		+	-	+	+	+	-	-	-	-	+	38	1
	2a	+	-	+	+	-	-	-	-	-	+	4	1
3		+	-	+	+	-	+	-	-	-	+	6	1
4		+	-	+	-	+	+	-	-	-	+	6	1
	4a	+	-	+	-	-	+	-	-	-	+	3	1
5		+	-	+	+	+	+	-	-	+	+	10	1
	5a	+	-	+	+	-	+	-	-	+	+	6	1
6		+	-	+	+	+	+	-	+	-	+	2	4
7		+	-	+	+	+	+	-	-	-	-	4	1
8		+	-	-	+	+	+	-	-	-	+	0	1
	8a	+	-	-	+	+	-	-	-	-	+	3	1
	8b	+	-	-	+	+	+	-	-	+	+	2	1
	8c	+	-	-	+	+	-	-	-	+	+	2	1
9		+	-	+	+	+	-	-	-	+	+	12	1
	9a	-	-	+	+	+	-	-	-	+	+	1	1
10		+	-	+	+	+	-	-	+	-	+	1	4
11		+	-	+	+	+	-	+	-	-	+	1	1
12		+	-	+	+	+	+	-	+	+	+	1	4
13		-	+	+	+	+	+	-	-	-	+	2	1
	13a	-	+	+	+	-	+	-	-	-	+	3	1
	13b	-	+	+	-	+	+	-	-	-	+	1	1
	13c	-	-	+	+	+	+	-	-	-	+	1	1
14		+	-	+	-	+	-	-	-	+	+	4	1
	14a	+	-	+	-	+	-	-	-	-	+	1	1
15		+	-	+	+	+	+	+	+	+	+	6	3
New Biogroup	16	+	-	+	+	+	+	+	-	+	+	6	2
	16a	+	-	+	+	-	+	+	-	+	+	1	2
	16b	+	-	+	+	+	+	+	-	-	+	1	2
	16c	+	-	+	-	-	+	+	-	+	+	1	2

^a^VP, Voges-Proskauer; MR, methyl red; Nit, nitrate reduction; Orn, ornithine utilization; Mot, motility at 37°C; Ino, acid production from inositol; Dul, acid production from dulcitol; Ind, indole production; Malo, malonate utilization; Gas, gas production from glucose. All isolates positively reacted with α-methyl-D-glu, with the exception of biogroup 15 isolates.

**Table 3:** Representative strains from each of the biogroups used in this study.

Biogroup	NTU^a ^strain number	Source	Origin
1	1	NCTC 11467^b^	Child's throat (Type strain) (ATCC 29544 = CDC 4562-70)
2	12	Cèské Budéjovice^c^	Faeces (adult 3753)
2a	512	Radboud^d^	Clinical (Prague 26248)
3	680	CDC 996-77^e^	Spinal fluid (New York, USA)
4	683	CDC 407-77	Sputum (Pennsylvania, USA)
4a	515	Radboud	Clinical (Prague 26706)
5	685	CDC 1716-77	Blood (Connecticut, USA)
5a	472	NTU	Infant formula (Korea)
6	564	CDC 5960-70	Blood (Florida, USA)
7	686	CDC 9369-75	Unknown
8a	22	FSM E321^f^	Infant formula (France)
8b	15	Cèské Budéjovice	Faeces (adult 2422)
8c	35	NTU	Infant cereal (Washington, USA)
9	681	CDC 1058-77	Breast abscess
9a	521	Radboud	Clinical (Prague 27288)
10	582	NCTC 9844	Unknown
11	684	CDC 3128-77	Sputum (Alabama, USA)
12	563	CDC 0743-75	Foot wound (Wisconsin, USA)
13	567	CDC 9363-75	Unknown
13a	535	Radboud	Milk powder (New Zealand)
13b	507	Radboud	Clinical (Prague 25569)
13c	101	NTU	Whole mixed spice
14	565	CDC 1895-73	Faeces (Maryland, USA)
14a	33	NTU	Baby rice (Herndon, USA)
15	566	CDC 3523-75	Bone marrow (Arizona, USA)
16	9	NTU	Infant food (cheese puffs)
16a	92	NTU	Herb (biota tops)
16b	57	NTU	Milk powder (UK)
16c	581	NCTC 9529	Water

^a^Nottingham Trent University, Nottingham, UK; ^b^NCTC, London, UK; ^c^Hospital Cèské Budéjovice, Czech Republic; ^d^Department of Medical Microbiology, Radboud, Nijmegen, Netherlands; ^e^CDC, Atlanta, GA, USA; ^f^Nestlé Research Center, Lausanne, Switzerland.

**Table 4:** Biochemical differentiation of *E. sakazakii *16S rDNA clusters.

Farmer Biogroup	Phenotype ^a^			No. of strains	Genomic cluster
			
	Ino	Dul	Ind		
1–5, 7–9, 13, 14	(+/-)	-	-	169	1
11	-	+	-	1	1
16	+	+	-	9	2
15	+	+	+	6	3
6, 10, 12	(+/-)	-	+	4	4

^a^Ino, acid production from inositol; Dul, acid production from dulcitol; Ind, indole production; (+/-), some stains positive, some strains negative, the test is used to define subgroups.
